# Diagnostic Yield of Combined Pulmonary Cryobiopsies and Electromagnetic Navigation in Small Pulmonary Nodules

**DOI:** 10.1155/2018/6032974

**Published:** 2018-11-15

**Authors:** Olivier Taton, Benjamin Bondue, Pierre Alain Gevenois, Myriam Remmelink, Dimitri Leduc

**Affiliations:** ^1^Department of Pneumology, Hôpital Erasme, Université Libre de Bruxelles, 808 Route de Lennik, 1070 Brussels, Belgium; ^2^Department of Radiology, Hôpital Erasme, Université Libre de Bruxelles, 808 Route de Lennik, 1070 Brussels, Belgium; ^3^Department of Pathology, Hôpital Erasme, Université Libre de Bruxelles, 808 Route de Lennik, 1070 Brussels, Belgium

## Abstract

**Background:**

An increasing number of pulmonary nodules of unknown nature are detected as a result of screening by CT in high lung cancer risk patients.

**Objectives:**

The purposes of this study were to assess the diagnostic yield of electromagnetic navigation bronchoscopy (ENB) combined with transbronchial lung cryobiopsy (TBLC) and to compare it with standard transbronchial biopsy (TBB) in pulmonary nodules of less than 2 cm in diameter.

**Methods:**

We prospectively included 32 patients (18 men and 14 women, mean age 68 ± 9 years) with nodules of less than 2 cm in diameter and no metastasis at ^18^FDG PET-CT. The nodule position was determined by ENB, radial endobronchial ultrasonography miniprobe, and fluoroscopy. Eight samples were obtained, six by TBB and two by TBLC.

**Results:**

Nodule diameter averaged 16 ± 3 mm. Twenty-five nodules were malignant and 18 were surgically resected. Surgery was avoided in four patients as the biopsies revealed a benign disease. The samples obtained by TBLC were five times larger than those by TBB. The diagnostic yields of TBLC and TBB were 69% and 38%, respectively (*p*=0.017). Adverse events consisted in 15 mild or moderate bleedings and one pneumothorax.

**Conclusions:**

In the setting of peripheral pulmonary lesions of less than 20 mm in diameter, ENB-combined TBLC is feasible and safe, provides larger samples, and has higher diagnostic yield than TBB.

## 1. Introduction

Since the report of decreased lung cancer mortality associated with screening trials in subjects at high lung cancer risk [1], the number of chest CT scans performed for the detection of lung cancer increased dramatically. As a consequence, an increasing number of solid and nonsolid (ground glass) nodules of unknown etiology are detected [2]. The challenge for the physician is to determine safely and accurately the pathological nature of these nodules.

It is acknowledged that flexible bronchoscopy under fluoroscopic guidance has a diagnostic yield lower than 30% for nodules less than 2 cm in diameter as compared to higher than 60% in those larger than 2 cm in diameter [3]. Interestingly, Eberhardt et al. [4] reported that the diagnostic yield of bronchoscopy could be improved up to 88% by combining electromagnetic navigation bronchoscopy (ENB) with endobronchial ultrasonography (EBUS) in order to guide transbronchial biopsy (TBB). However, their study group included patients with a wide range of nodule sizes with only 23 patients with nodules smaller than 2 cm in diameter.

On the other hand, the size of tissue samples obtained with standard forceps usually limits the diagnostic yield in small nodules especially in the era of immunohistochemical and molecular testing required for lung cancer targeted therapies [5]. Transbronchial lung cryobiopsy (TBLC) is an emerging technique that allows tissue samples larger than those obtained with standard TBB in the setting of interstitial lung diseases [6, 7] and has been shown to be feasible under EBUS guidance in the setting of pulmonary nodules [8, 9]. We thus hypothesized that TBLC could perform better than TBB in the workup of nodules smaller than 2 cm in diameter. The purpose of this prospective study was therefore to compare the diagnostic yield of TBLC and TBB, both guided by an EBUS miniprobe combined with ENB, in this particular setting.

## 2. Materials and Methods

### 2.1. Study Design and Patients

This study protocol was approved by our institutional ethics committee and written informed consent was obtained from all patients.

**Figure 1:**
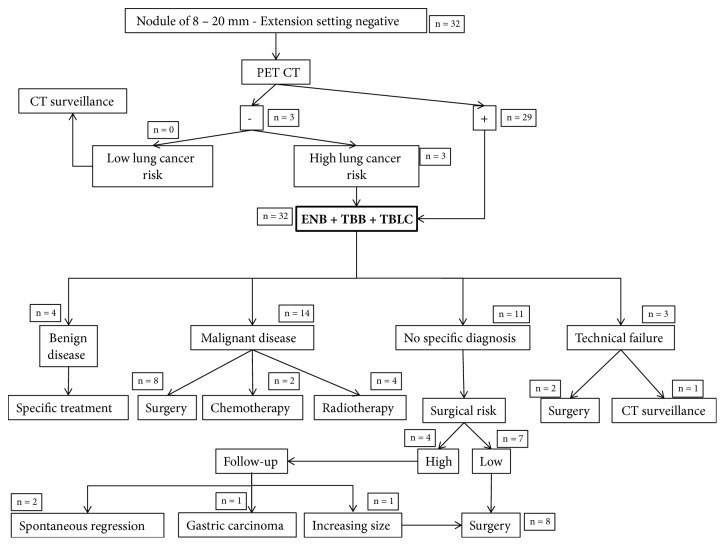
Flowchart summarizing the management of patients with pulmonary nodules from 8 to 20 mm in diameter without locoregional or distant metastasis with the frequencies in each category. Benign diseases consisted in tuberculosis in three patients, sarcoidosis in one patient, and cryptogenic organizing pneumonia in one patient. Abbreviations: ENB = electromagnetic navigation bronchoscopy; EBUS = endobronchial ultrasonography; TBB = transbronchial biopsy; TBLC = transbronchial lung cryobiopsy.

From December 2016 to March 2018, 32 consecutive patients (18 men and 14 women, mean age (± standard deviation, SD) 68 ± 9 years) were prospectively enrolled. Twelve patients were current smokers and 17 patients were ex-smokers. On average, the smokers had smoked 37 ± 23 pack-years. All patients underwent chest CT scan the day before bronchoscopy. The location and the size of each nodule were recorded. Patients with pulmonary solid or nonsolid nodules were considered for inclusion in our study group if they fulfilled the following criteria: age over 18 years; a CT-detected solid or nonsolid nodule with a diameter ranging from 8 to 20 mm without any evidence of locoregional or distant metastasis; no endobronchial abnormality; platelets count higher than 80,000/mm^3^; systolic pulmonary arterial pressure lower than 45 mmHg at transthoracic ultrasonography; no coagulation tests abnormality; no severe respiratory dysfunction defined as FEV_1_ < 1L, FVC < 50% predicted, or DLCO < 25% predicted; and no contraindication to general anesthesia or bronchoscopy. Metabolic activity of lung nodules was assessed by a ^18^FDG PET-CT scan. In absence of ^18^FDG uptake, patients at high lung cancer risk (age of 55 to 74 years, more than 30 pack-years of smoking history, growing lung lesion, or neoplasm history) were also included.  Figure 1 summarizes the management of these patients.

### 2.2. Tissue Sampling

General anesthesia and muscles paralysis were obtained with remifentanil (Ultiva®, Aspen Pharma, Dublin, Ireland), propofol (Propolipid®, Fresenius Kabi, Hesse, Germany), and rocuronium (Esmeron®, MSD, Kenilworth, NJ). After insertion of a rigid bronchoscope, a guide sheath (Edge™ Firm Tip, endobronchial procedure kit, 180° catheter, Covidien, MA, USA) was first inserted through a flexible bronchoscope (BF-1TH190, Olympus, Tokyo, Japan) in the segmental bronchus close to the target nodule with the previously described ENB technique (superDimension/Bronchus, Herzliya, Israel) [10]. The ENB was considered successful if the distance from the sensor probe to the target center was less than 10 mm according to Becker et al. [10]. The average fiducial target registration error, which is the discrepancy between the virtual and real bronchoscopy, was not assessed in this study. A radial EBUS miniprobe (UM-S20-17S, 20 MHz, Olympus, Tokyo, Japan) was then inserted in the guide sheath in order to check its position close to the target nodule. Finally, the position holding of the catheter was checked at regular time intervals by fluoroscopy.

Six samples were obtained through the bronchial wall (i.e., TBB) with a standard pair of forceps (FB-233D, 3 Fr, Olympus, Tokyo, Japan), the position holding of the catheter having been checked by ENB after the three first samples. All the material was then removed in order to place a Fogarty balloon into the segmental bronchus, which was inflated prophylactically after each cryobiopsy for controlling possible bleeding following TBLC. The ENB procedure was performed again. A first TBLC sample was obtained through a flexible cryoprobe of 115 cm in length and 1.9 mm in diameter (ERBE, Medizintechnik GmbH, Tubingen, Germany) inserted into the guide sheath. Once in position, the probe was cooled for 7 to 8 seconds; then the guide sheath, the cryoprobe, and the bronchoscope were together removed out of the airway and the frozen specimen was thawed first in saline at room temperature and afterwards transferred to formalin for fixation [6]. A second TBLC sample was obtained if no significant complication was observed. ENB technique and fluoroscopy were performed before each TBLC in order to position again the guide sheath. Adverse events occurring during and after completion of the procedure were recorded.

Among possible complications, bleeding was scored as follows: score 0, when no bleeding occurred; score 1, when bleeding stopped within five minutes either spontaneously or by inflation of the Fogarty balloon; score 2, when bleeding was prolonged for more than five minutes or needed cold saline instillation; and score 3, when bleeding required embolization, selective bronchial intubation, transfusion, or admission in the intensive care unit or resulted in prolonged hospital stay or patient's death [6].

All samples were analyzed by a pathologist (blind for review) with more than 25 years of experience in lung pathology. If the diagnosis based on TBB and TBLC samples was uncertain or inconsistent with the clinical presentation, operable patients were referred for surgical resection and nonoperable patients were followed up by CT six months thereafter. Depending on histological analyses, microbiological analyses (i.e., specific staining and culture) were requested if needed. Pathological analysis of the surgical specimen and CT follow-up were considered as the independent methods of reference for establishing the final diagnosis.

### 2.3. Statistics

Continuous data were expressed using means ± SD. Categorical data were expressed as percentages. The diagnostic yield of each technique is the ratio of correctly diagnosed cases to the total number of patients expressed in percentage. Comparisons between groups were performed using the chi-square or Fisher's exact test when appropriate. A two-tailed* p* value of less than 0.05 was considered as statistically significant. Statistical analysis was performed using SAS (SAS Institute, Cary, NC).

## 3. Results

### 3.1. Patients and Nodules Characteristics

**Figure 2:**
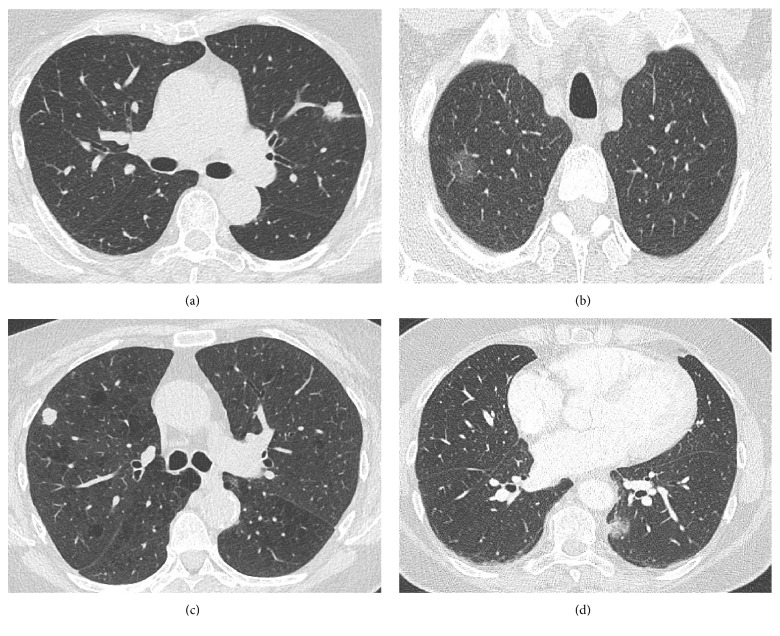
CT scans in four representative patients. Panel (a) presents a solid nodule of 16 mm in diameter in the left upper lobe in a 75-year-old woman. The final diagnosis was tuberculosis. Panel (b) presents a nonsolid nodule of 20 mm in diameter in the right upper lobe in a 57-year-old man. The final diagnosis was lung adenocarcinoma. Panel (c) presents a solid nodule of 11 mm in diameter in the right upper lobe in a 72-year-old woman. The final diagnosis was small cell lung carcinoma. Panel (d) presents a nonsolid nodule of 14 mm in diameter in the left lower lobe in a 67-year-old woman. The final diagnosis was lung adenocarcinoma.

Patients and nodules characteristics are summarized in Table 1. The mean diameter of the target nodules was 16 ± 3 mm. All patients but one presented one single nodule. One patient presented four nodules. In that particular patient, the largest nodule (16 mm in diameter) was selected for biopsy. All nodules were solid but one was nonsolid. Fifteen, sixteen, and one patient, respectively, had the nodule in their upper, lower, or middle lobe. Eleven patients presented a bronchus sign at CT [11].  Figure 2 showed 4 representative patients in our study group.

Fifteen patients were operable at the time of inclusion according to the ERS guidelines [12] but preferred to have first the endoscopic evaluation before a surgical procedure. At the end of this study, eleven among these 15 operable patients had surgery that revealed a malignant disease and surgery was avoided in four patients as the endoscopic procedures could yield the diagnosis of a benign disease.

### 3.2. Final Diagnosis

Malignancy was diagnosed in 25 patients. Among these 25 patients, 20 presented a lung adenocarcinoma, two a squamous cell cancer, two a small cell lung cancer, and one a metastasis of a gastric carcinoma. The diagnosis was confirmed by surgery in 18 patients and by TBLC/TBB in six patients. In one patient, a gastric carcinoma was diagnosed by gastric biopsy during the follow-up and the pulmonary nodule was assumed to be a lung metastasis from the gastric carcinoma. Other diagnoses included tuberculosis in three patients, nonspecific inflammation with spontaneous regression in two patients, cryptogenic organizing pneumonia in one patient, and sarcoidosis in one patient.

### 3.3. Technical Results

In three patients, we were unable to reach the target with the ENB. As a consequence, the method was successful in 29 patients. Among these 29 patients, the nodule was visualized at fluoroscopy in eight patients and at EBUS miniprobe in 19 patients.

### 3.4. Comparison between TBLC and TBB

The mean diameters of the samples, respectively, obtained by TBLC and TBB were 5.3 mm ± 0.7 and 1.1 mm ± 0.6 (*p*< 0.001) (Table 2).

Among the 29 patients in whom both TBLC and TBB could be obtained, their overall diagnostic yield was 69% (20/29) and 38% (11/29), respectively (*p*=0.017). Considering the three patients in whom the target nodule could not be reached with ENB, the diagnostic yield was 63% (20/32) and 34% (11/32), respectively (*p*=0.024).

Among the 29 patients in whom both TBLC and TBB could be obtained, the sensitivity and specificity of TBLC for the diagnosis of a malignant nodule were, respectively, 61% and 100% as compared to 35% and 100% for TBB (*p*=0.008 and* p*>0.999, respectively). The corresponding positive and negative predictive values of TBLC were, respectively, 100% and 40% as compared to 100% and 29% for TBB (*p*>0.999 and* p*=0.277, respectively).

The lobar location of the nodule, the bronchus sign, the nodule size, the malignant vs. benign disease, or the technique used (nodule visualization or not with EBUS miniprobe) for visualizing the nodule in addition to ENB had no statically significant impact on the diagnostic performance (*p* ranging from 0.073 to 0.934) (Table 3).

We have also calculated the number needed to be tested (by endoscopic procedure) to diagnose benign disease and we obtained the number of 8. It means that we have to perform 8 endoscopic procedures to diagnose one benign disease and to avoid an inadequate treatment such as surgical resection or stereotactic ablative radiotherapy.

### 3.5. Adverse Events

Bleeding was graded 1 and 2 in, respectively, eleven and four patients. Pneumothorax needing pleural drainage for three days was observed in one patient. No other adverse event was observed and no mortality was recorded.

## 4. Discussion

This study shows that, in the workup of pulmonary nodules smaller than 2 cm in diameter, TBLC performs better than TBB and is safe with no or minimal associated adverse events.

We used TBLC in order to obtain as large as possible tissue samples and we combined it with fluoroscopy, EBUS miniprobe, and ENB in order to accurately reach the target nodules. With this strategy, the overall diagnostic yield of TBLC reaches 63%, a figure in line with results reported by Wang et al. in their meta-analysis also focused on lesions smaller than 20 mm in diameter (60.9% with a 95% confidence interval ranging from 54.0 to 67.7%) [13].

As compared to TBB, TBLC provides larger tissue samples and has also the theoretical advantage of reaching nodules adjacent to bronchus segments between successive divisions that are inaccessible by TBB as it requires that the pair of forceps faces the target nodule. The difference of diagnostic yields between TBLC and TBB—respectively, around 70% and 40%—confirms these advantages. As previously reported [7], the samples obtained by TBLC are indeed approximately five times larger than those by TBB. This larger size could probably explain that the diagnostic yield and sensitivity of TBLC are higher than those of TBB in lung nodules. However, the sensitivity and the negative predictive value remain low with prevalence of malignancy approximating 80% in our study group, very close to that reported by Gex et al. (76.5% with a 95% confidence interval ranging from 70.2% to 81.2%) in their systematic review [14].

TBLC associated with ENB could be the best option in the diagnosis of nodules smaller than 20 mm in diameter. CT-guided transthoracic needle biopsy is an alternative to assess peripheral lesions but is associated with a higher complication rate than endoscopy [15], especially in lesions smaller than 30 mm in diameter, in which the rate of pneumothorax reaches 32.3% [16]. In our study group, the rate of bleeding and pneumothorax was particularly low, rising the interest of TBLC associated with ENB in the particular setting of small-size nodules. Indeed, we observed lower bleeding—with no severe bleeding—and pneumothorax rates than in the setting of interstitial lung disease [17]. The only patient who required chest tube drainage had a nodule adjacent to the pleura, suggesting avoidance of cryobiopsy in this particular location.

In order to improve the accuracy of lesion targeting, new guidance techniques have been developed. However, despite the use of three complementary localization techniques (fluoroscopy, EBUS miniprobe, and ENB), the diagnostic yield of TBB in our study was much lower than 65% and 67% reported in two recent systematic reviews [10, 13]. Our low diagnostic yield could be explained, at least in part, by the recruitment in our tertiary hospital of more challenging cases, including small targets, a very low frequency of the CT bronchus sign, a sign considered as a key variable conditioning ENB yield with an odd's ratio averaging 7.6 [11], and a low proportion of nodules visualized with the EBUS miniprobe, such visualization being also associated with an increased yield [4].

Whereas surgical resection is the currently recommended treatment in operable patients with lung nodule, high lung cancer risk, and no metastasis [18], 15 of our patients preferred to have first an endoscopic evaluation. Among them, four patients presented a benign disease and surgery could thus be avoided. In five other patients, malignancy was proved by an endoscopic procedure and they were operated on. The six remaining patients had no specific diagnosis after endoscopic procedure and were also operated on with a subsequent diagnosis of lung cancer in all of them. The actual benefit of endoscopic procedures appears thus lower in operable patients than in nonoperable ones but could nevertheless be an option in selected patients and could be discussed with them. On the other hand for nonoperable patients, stereotactic ablative radiotherapy has proven efficacy in the control of early stage cancer with few adverse events [19]. Considering this locoregional treatment and systemic treatment currently available, in nonoperable patients or in patients with intermediate lung cancer risk (for example, nongrowing or PET-negative nodules), cryobiopsy could be considered in order to propose specific treatments. We could also speculate that combining endoscopic approaches (ENB, EBUS miniprobe, and fluoroscopy), TBLC, and rapid on-site pathological examination followed by surgery would be an interesting future option to investigate.

Our study has two important limitations. First, the small number of included patients precludes definite conclusions on the appropriateness of TBLC in small lung nodules. Second, this is a monocentric study limiting the impact of procedural variability. Our results should therefore encourage further randomized and multicentric studies with a standardized approach of the nodules of less than 20 mm in diameter.

In conclusion, in the setting of peripheral pulmonary lesions of less than 20 mm in diameter, ENB-combined TBLC is feasible and safe, provides larger tissue samples, and has higher diagnostic yield than TBB. This approach could be proposed in nonoperable or low/intermediate lung cancer risk patients.

## Figures and Tables

**Table 1:** Patients characteristics.

Patients characteristics at inclusion in the study	

Male gender (%)	56 (18/32)
Age (yrs)	68 ± 9
Current smoker (%)	37 (12/32)
Previous smoker (%)	53 (17/32)
Pack-year	37 ± 23
Body height (cm)	169 ± 8
Body weight (kg)	73 ± 15
FEV_1_ (ml)	1851 ± 716
FEV_1_ (% predicted)	71.5 ± 20.1
DLCO (% predicted)	58.8 ± 19.2
PAPs (mmHg)	30 ± 5
Lesion size (mm)	16 ± 3

Lobar distribution of nodules at initial CT scan	

Right upper lobe	8
Middle lobe	1
Right lower lobe	8
Left upper lobe	7
Left lower lobe	8

Malignancy at the end of follow-up	

Lung adenocarcinoma	20
Squamous cell cancer	2
Small cell lung cancer	2
Metastatic carcinoma	1

Note: data are presented as frequencies or means ± SD. Abbreviations: FEV_1_ = forced expiration volume in 1 second; DLCO = carbon monoxide diffusing capacity; PAPs = systolic pressure of pulmonary artery.

**Table 2:** Comparison between TBB and TBLC in 29 patients in whom both TBLC and TBB were obtained.

	TBB	TBLC	*p* value
Sample size (mm)	1.1 ± 0.6	5.3 ± 0.7	<0.001
Diagnostic yield *∗*	38 % (11/29)	69 % (20/29)	0.017
Sensitivity	35%	61%	0.008
Specificity	100%	100%	>0.999
Positive PV	100%	100%	>0.999
Negative PV	29%	40%	0.277
Bleeding			
Grade 1	7%	38%	0.005
Grade 2	0%	14%	0.043
Grade 3	0%	0%	>0.999

Note: abbreviations: TBB = transbronchial biopsy; TBLC = transbronchial lung cryobiopsy; PV = predictive value. *∗*: diagnosis was obtained by TBLC alone in 9 patients and by both TBB and TBLC in 11 patients.

**Table 3:** Comparison between diagnosis accuracy by TBB and that by TBLC.

	< 15 mm	≥ 15 mm	*p*
TBB	5/11 (45 %)	6/18 (33 %)	0,540
TBLC	6/11 (54 %)	14/18 (78 %)	0,230

	Lower lobes	Other lobes	

TBB	6/15 (40 %)	5/14 (36 %)	0,820
TBLC	10/15 (67 %)	10/14 (71 %)	0,791

	Malignant disease	Benign disease	

TBB	8/23 (35 %)	3/6 (50 %)	0,555
TBLC	14/23 (61 %)	4/6 (67 %)	0,73

	EBUS +	EBUS -	

TBB	7/19 (37 %)	4/10 (40 %)	0,876
TBLC	13/19 (68 %)	7/10 (70 %)	0,934

	Bronchus sign +	Bronchus sign -	

TBB	3/11 (27 %)	8/18 (44 %)	0,329
TBLC	8/11 (73 %)	12/18 (67 %)	0,816

Note: abbreviations: TBB = transbronchial biopsy; TBLC = transbronchial lung cryobiopsy; EBUS = endobronchial ultrasonography; EBUS + = nodule visualization by EBUS miniprobe. EBUS - = no nodule visualization by EBUS miniprobe. Bronchus sign + = presence of a bronchus sign. Bronchus sign - = absence of bronchus sign.

## Data Availability

The chest CT scans, PET-CT scans, pathological analysis, endoscopic procedure, and patients' data used to support the findings of this study are available from the corresponding author upon request.

## Abbreviations

DLCO: Diffusing capacity for carbon monoxide
EBUS: Endobronchial ultrasonography
ENB: Electromagnetic navigation bronchoscopy
FEV_1_: Forced expiration volume in one second
FVC: Forced vital capacity
TBB: Transbronchial biopsy
TBLC: Transbronchial lung cryobiopsy.
